# Supplementary material for: Six-Month Follow-Up after Vaccination with BNT162b2: SARS-CoV-2 Antigen-Specific Cellular and Humoral Immune Responses in Hemodialysis Patients and Kidney Transplant Recipients
Source: Pathogens. 2022 Jan 5;11(1):67. doi: 10.3390/pathogens11010067 (PMC8780885; doi:10.3390/pathogens11010067)
Supplement: Supplementary file 1 [file pathogens-11-00067-s001.zip › pathogens-1526363-supplementary.pdf]

*Supplementary Materials*

*Table S1.: **Number of patients** per study visit and divided into age and sex*

| Visit | | KTR | HDP | control |
|---|---|---|---|---|
| **V1** | **total** | **39** | **152** | **19** |
| | **based on sex** | female: 16; male: 23 | female: 52; male: 100 | female: 14; male: 5 |
| | **based on age** | <40: 10y; 40 – 60y: 19; >60y: 10 | <40y: 10; 40 – 60y: 29; 60 – 80y: 85; > 80y: 28 | <40y: 2; 40 – 60y: 10; >60y: 7 |
| **V2** | **total** | **43** | **147** | **19** |
| | **based on sex** | female: 16; male: 27 | female: 54; male: 93 | female: 14; male: 5 |
| | **based on age** | <40y: 9; 40 – 60y: 23; >60y: 11 | <40y: 9; 40 – 60y: 34; 60 – 80y: 80; > 80y: 24 | <40y: 3; 40 – 60y: 10; >60y: 6 |
| **V3** | **total** | **29** | **156** | **22** |
| | **based on sex** | female: 10; male: 19 | female: 54; male: 102 | female: 14; male: 8 |
| | **based on age** | <40y: 6; 40 – 60y: 17; >60y: 6 | <40y: 8; 40 – 60y: 35, 60 – 80y: 85; > 80y: 28 | <40y: 4; 40 – 60y: 12; >60y: 6 |

***Table S2: Mapping of HLA genotype in association with humoral vaccination response in 33 KTR.*** *The following table shows the HLA genotype of 33 KTR patients currently on the waiting list for kidney transplantation. In red are the HLA alleles that have been associated with severe COVID-19 disease progression in Europe. In our data, there is no association with a lack of or weak humoral vaccine response and the known alleles leading to more severe COVID-19. Rather, the results can be related to the strength of immunosuppression. However, due to the small number of patients, the data are limited.*

| patient | HLA-genotyp | | | | | vaccination response | | | |
|---|---|---|---|---|---|---|---|---|---|
| | A | B | C | DR | DQ | **IgG anti-S1 (AU/ml) V1** | **Ig total (U/ml) V1** | **IgG anti-S1 (AU/ml) V2** | **Ig total (U/ml) V2** |
| **KTR 1** | A*01, A*03 | B*44, B*35 | C*04, C*05 | DRB1*12, DRB1*13 | DQB1*06, DQB1*03 | 1,2 | 0,4 | 152,7 | 9,49 |
| **KTR 2** | A*03 | B*44, B*40 | C*03, C*05 | DRB1*12, DRB1*13 | DQB1*06, DQB1*03:01 | 4,8 | 0,4 | 4,8 | - |
| **KTR 3** | A*01, A*02 | B*07, B*08 | C*07 | DRB1*15, DRB1*03:01 | DQB1*06, DQB1*02 | 0 | 0,4 | 6,3 | 0,4 |
| **KTR 4** | A*02, A*29 | B*44, B*15 | C*03, C*16 | DRB1*04, DRB1*13 | DQB1*06, DQB1*03:02 | 0 | 0,4 | 0 | 0,4 |
| **KTR 5** | A*03, A*11 | B*38, B*40 | C*02, C*12 | DRB1*11, DRB1*13 | DQB1*06, DQB1*03:01 | 0 | - | 0 | 0,4 |

| | | | | | | | | | |
|---|---|---|---|---|---|---|---|---|---|
| **KTR 6** | A*24, A*32 | B*55, B61 | C*02, C*03 | DRB1*16, DRB1*13 | DQB1*05, DQB1*06 | 3,8 | 0,4 | 2,4 | 0,4 |
| **KTR 7** | A*03, A*24 | B*08, B*55 | C*03, C*07 | DRB1*01, DRB1*03:01 | DQB1*05, DQB1*02 | 0 | 0,4 | 11,8 | 0,4 |
| **KTR 8** | A*30, A*32 | B1*3, B*39 | C*w6 | DR*11 DR*8 | DQ3, DQ4 | - | - | 0,0 | 0,4 |
| **KTR 9** | A*01, A*03 | B*08, B*35 | C*04, C*07 | DRB1*03:01, DRB1*13 | DQB1*06, DQB1*02 | 0 | 0,4 | 0,3 | 0,4 |
| **KTR 10** | A*02 | B*27, B*40 | C*02, C*03 | DRB1*04, DRB1*11 | DQB1*03 | 32 | 0,4 | - | - |
| **KTR 11** | A*01, A*24 | B*08, B*18 | C*07 | DRB1*15, DRB1*03:01 | DQB1*06, DQB1*02 | 0 | 0,4 | 0,8 | 0,4 |
| **KTR 12** | A*02, A*68 | B*15, B*40 | C*03, C*07 | DRB1*04, DRB1*13 | DQB1*03 | 27,5 | 0,4 | 16,2 | 0,4 |
| **KTR 13** | A*01, A*24 | B*39, B*27 | C*02, C*07 | DRB1*01, DRB1*04 | DQB1*05, DQB1*03:02 | 2,5 | 0,4 | - | - |
| **KTR 14** | A*01, A*02 | B*08, B*57 | C*06, C*07 | DRB1*15, DRB1*03:01 | DQB1*06, DQB1*02 | 4,5 | 0,4 | 3,6 | 0,4 |
| **KTR 15** | A*32, A*33 | B*13, B*18 | C*02, C*06 | DRB1*04, DRB1*07 | DQB1*02, DQB1*03:02 | 2,4 | 0,4 | 0 | 0,4 |
| **KTR 16** | A*30, A*32 | B*18, B*35 | C*04, C*05 | DRB1*03:01, DRB1*13 | DQB1*06, DQB1*02 | 1,5 | 0,4 | 121,7 | 26,09 |
| **KTR 17** | A*1, A*66 | B*8, B*41 | Cw7 | DRB1*17, DRB1*13 | DQB1*06, DQB1*02 | 0 | 0,4 | - | - |
| **KTR 18** | A*02, A*03 | B*35, B*40 | C*03, C*04 | DRB1*01, DRB1*16 | DQB1*05 | - | - | 1,2 | 0,4 |
| **KTR 19** | A*02 | B*57, B*40 | C*03, C*06 | DRB1*13, DRB1*07 | DQB1*06, DQB1*02 | - | - | 2,2 | 0,4 |
| **KTR 20** | A*03 | B*51, B*57 | C*06, C*14 | DRB1*11, DRB1*07 | DQB1*03:01, DQB1*03:03 | 0 | 0,4 | - | - |
| **KTR 21** | A*01, A*02 | B*44, B*39 | C*05, C*07 | DRB1*11, DRB1*08 | DQB1*03, DQB1*04 | | | 833,6 | 39,44 |
| **KTR 22** | A*01, A*68 | B*51, B*3 | C*06, C*07 | DRB1*16, DRB1*13 | DQB1*05, DQB1*06 | 2,7 | 0,4 | 14,8 | 0,4 |
| **KTR 23** | A*02, A*24 | B*15, B*57 | C*03, C*06 | DRB1*04, DRB1*13 | DQB1*06, DQB1*03 | 22,2 | 0,4 | 72,4 | 12,08 |
| **KTR 24** | A*02 | B*07, B*35 | C*04, C*07 | DRB1*01, DRB1*15 | DQB1*05, DQB1*06 | 1,8 | 0,4 | - | - |
| **KTR 25** | A*02, A*24 | B*14, B*35 | C*04, C*08 | DRB1*13, DRB1*14 | DQB1*05, DQB1*03 | - | - | 3156,3 | >250 |
| **KTR 26** | A*02, A*32 | B*13, B*50 | C*06 | DRB1*07, DRB1 *4 | DQB1*02 | 1,5 | 0,4 | 1,5 | 0,4 |
| **KTR 27** | A*03, A*32 | B*18, B*56 | C*01, C*07 | DRB1*11, DRB1*14 | DQB1*05, DQB1*03:01 | 1,1 | 0,4 | 2,2 | 0,506 |
| **KTR 28** | A*02, A*26 | B*38, B*40 | C*12 | DRB1*15, DRB1*04 | DQB1*06 DQB1*03 | 62,6 | 3,12 | 1750,6 | >250 |
| **KTR 29** | A*01, A*23 | B*53, B*81 | C*04, C*18 | DRB1*13, DRB1*07 | DQB1*02 | 0,2 | 0,4 | 28,5 | 0,4 |
| **KTR 30** | A*01, A*24 | B*08 | C*07 | DRB1*03, DRB1*04 | DQB1*02, DQB1*03 | 0,1 | 0,4 | 0,4 | 0,4 |
| **KTR 31** | A*01, A*24 | B*07, B*37 | C*06, C*07 | DRB1*14, DRB1*10 | DQB1*05 | 0,6 | 0,4 | 2,2 | 0,4 |
| **KTR 32** | A*24, A*66 | B*51, B*44 | C*01, C*12 | DRB1*01, DRB1*07 | DQB1*05, DQB1*02 | - | - | 4,5 | 1,92 |
| **KTR 33** | A*01, A*03 | B*15, B*40 | C*01, C*03 | DRB1*07, DQB1*06 | DQB1*06, DQB1*02 | 1626,4 | 0,4 | 4663,1 | 250 |

***Table S3: Mapping of HLA genotype in association with humoral vaccination response in 15 HDP.*** *The following table shows the HLA genotype of 15 dialysis patients currently on the waiting list for kidney transplantation. In red are the HLA types that have been associated with severe COVID-19 disease progression in Europe. In our data, there is no correlation between a reduced vaccination response and the HLA characteristics that appear to be associated with more severe COVID-19 disease. Likewise, no association with individual other HLA types could be shown. However, the very small number of patients is limiting.*

| patient | HLA-genotyp | | | | | vaccination response | | | |
|---|---|---|---|---|---|---|---|---|---|
| | A | B | C | DR | DQ | IgG anti-S1 (AU/ml) V1 | Ig total (U/ml) V1 | IgG anti-S1 (AU/ml) V2 | Ig total (U/ml) V2 |
| **HDP 1** | A*02, A*66 | B*44, B*41 | C*05, C*17 | DRB1*15, DRB1*13 | DQB1*06, DQB1*03:01 | 3,4 | 0,4 | 12,01 | 1,05 |
| **HDP 2** | A*02, A*11 | B*51, B*44 | C*05, C*16 | DRB1*04, DRB1*14 | DQB1*05, DQB1*03:01 | 8,5 | 0,4 | 431,8 | 105,7 |
| **HDP 3** | A*02, A*11 | B*15, B*46 | C*01, C*08 | DRB1*12, DRB1*09 | DQB1*03:01, DQB1*03:03 | - | - | 37356 | 250 |
| **HDP 4** | A*02, A*11 | B*15, B60 | C*04, C*08 | DRB1*12, DRB1*14 | DQB1*05, DQB1*03:01 | - | - | 8568,7 | 250 |
| **HDP 5** | A*24, A*30 | B*13 | C*06 | DRB1*12, DRB1*07 | DQB1*03:01 | 95,9 | 2,11 | 4492,3 | >250 |
| **HDP 6** | A*03 | B*07, B*15 | C*03, C*07 | DRB1*15, DRB1*04 | DQB1*06, DQB1*03:01 | 129,7 | 8,53 | 4012,8 | >250 |
| **HDP 7** | A*02, A*03 | B*51, B*35 | C*04 | DRB1*01, DRB1*04 | DQB1*05, DQB1*03:01 | 765,5 | 56,26 | 4166,3 | >250 |
| **HDP 8** | A*02, A*11 | B*07, B*14 | C*07, C*08 | DRB1*15, DRB1*07 | DQB1*06, DQB1*02 | 230 | 11,73 | 2034,3 | >250 |
| **HDP 9** | A*24, A*11 | B*14, B*50 | C*06, C*08 | DRB1*01, DRB1*07 | DQB1*05, DQB1*02 | 27,9 | 0,789 | 1379,7 | >250 |
| **HDP 10** | A*02: A*03 | B*44, B*39 | C*05, C*12 | DRB1*04, DRB1*11 | DQB1*03:02 | 93,7 | 3,64 | 5169,5 | >250 |
| **HDP 11** | A*02 | B*18, B*40 | C*03, C*05 | DRB1*03:01, DRB1*13 | DQB1*06, DQB1*02 | 46,6 | 3,83 | - | - |
| **HDP 12** | A*03, A*68 | B*13, B*18 | C*05, C*06 | DRB1*03:01, DRB1*07 | DQB1*02 | 88,9 | 5,82 | - | - |
| **HDP 13** | A*02 | B*49, B60 | C*02, C*04 | DRB1*04, DRB1*11 | DQB1*03:01, DQB1*03:02 | 13,1 | 0,4 | 110,4 | 0,987 |
| **HDP 14** | A*02, A*68 | B*44, B*53 | C*02, C*04 | DRB1*04, DRB1*13 | DQB1*06, DQB1*03:02 | 0 | 0,4 | 2925,9 | >250 |
| **HDP 15** | A*02, A*03 | B*15, B*41 | Cw10 Cw17 | DRB1*04, DRB1*13 | DQB1*03:01, DQB1*03:02 | - | - | 7656,3 | >250 |

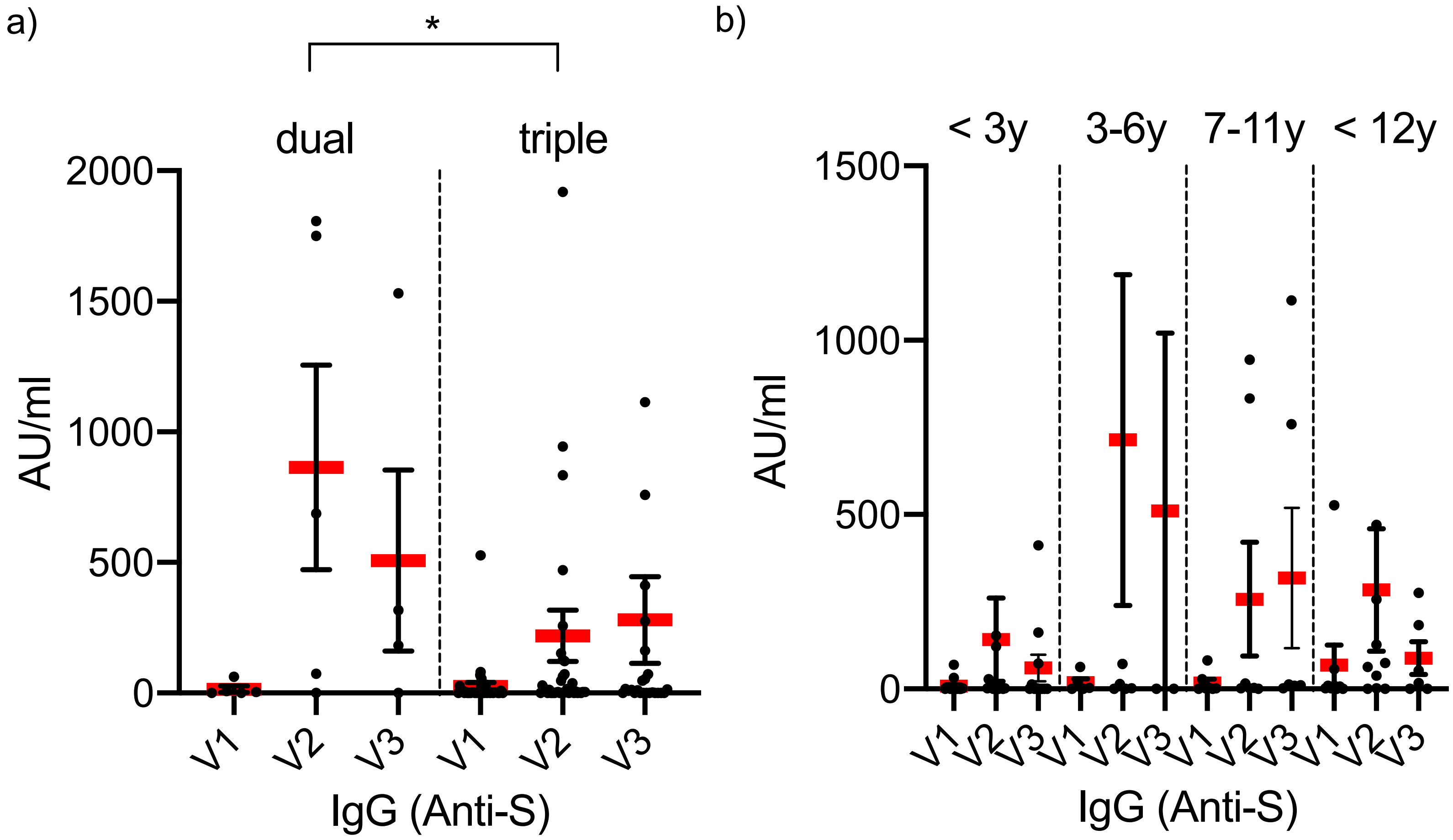


***Figure S1: Humoral vaccine response measured by SARS-CoV-2 specific anti-spike antibodies referred to immunosuppression and timepoint of transplantation in KTR.*** *<u>Figure S1 a)</u> shows the vaccination response measured by anti-spike (s) IgG antibodies referred to dual or triple immunosuppression regime at 1 month after the 1st vaccination (V1) and 1 month (V2) after the 2nd vaccination and 6 months (V3) after 1st vaccination. The x-axis represents the time points of measurement of vaccination response; the y-axis represents the anti-spike antibody titer in AU/ml. <u>Figure S1 b)</u> shows the vaccination response in KTR measured by anti-spike (s) IgG antibodies divided into how long*

*ago the kidney transplant was performed at the time of the vaccination control at V1, V2 and V3. The x-axis represents the time points of measurement of vaccination response; the y-axis represents the anto-spike antibody titer in AU/ml.*
